# Supplementary material for: Interleukin-6-Production Is Responsible for Induction of Hepatic Synthesis of Several Chemokines as Acute-Phase Mediators in Two Animal Models: Possible Significance for Interpretation of Laboratory Changes in Severely Ill Patients
Source: Biology (Basel). 2022 Mar 18;11(3):470. doi: 10.3390/biology11030470 (PMC8945369; doi:10.3390/biology11030470)
Supplement: Supplementary file 1 [file biology-11-00470-s001.zip › biology-1581318-supplementary.pdf]

Supplementary data

Table S1: Ct-values obtained from RT-PCR analysis of total liver RNA of WT and IL6-KO-mice compared to control mice. The mice were treated with turpentine oil (TO)-administered intramuscularly into the right and left hindlimb of mice at a concentration of 5 ml/kg/each hindlimb. Saline treated mice served as controls. Ct-values of GAPDH, CXCL1 and CXCL2, CXCL5, CXCL8 and CXCL10 are presented. Both treated and control animals were sacrificed at 2, 4, 6, 12 and 24 h after injections. Results represent mean ± SEM values of three experiments (in duplicate).

	**WT**	**IL6-KO**	**WT**	**IL6-KO**	**WT**	**IL6-KO**
Time (h)	GAPDH		CXCL1		CXCL2	
Control	15.1±0.3	15.4±0.2	29.1±0.3	29.1±0.5	33.9±0.3	34.3±1
2	15.0±0.3	15.4±0.1	22.9±0.2	29.1±0.3	33.8±0.2	34.9±1
4	16.4±0.2	15.8±0.2	23.6±0.1	28.7±0.3	33.5±1	35.4±0.7
6	15.1±0.4	16.5±0.4	22.3±0.2	28.9±0.8	31.4±0.4	33.5±0.3
12	15.0±0.3	15.2±0.2	21.9±0.2	28.5±0.6	29.3±0.7	32.4±0.6
24	15.2±0.1	15.1±0.1	22.3±0.6	28.1±0.4	31.2±0.6	34.0±0.5

	**WT**	**IL6-KO**	**WT**	**IL6-KO**	**WT**	**IL6-KO**
Time (h)	CXCL5		CXCL8		CXCL10	
Control	33.3±0.54	35.4±2.5	35.2±0.3	36.9±1.3	28.9±0.4	27.6±0.3
2	32.8±0.6	35.6±2	29.1±.2	37.7±1.4	27.8±0.2	28.4±0.2
4	32.4±0.4	36.1±0.1	29.8±0.01	35.9±1.5	30.2±0.4	29.2±0.2
6	32.7±0.4	37.2±0.9	28.6±0.1	33.9±0.3	29.5±0.2	30.9±0.8
12	29.1±1	38.4±1	28.2±0.1	35.6±0.5	30.1±0.3	28.6±0.06
24	30.2±1	34.1±1.2	29.4±0.4	33.2±0.4	28.5±0.2	29.2±0.2

Table S2: Ct-values obtained from RT-PCR analysis of total liver RNA of WT and IL6-KO-mice and compared with control mice. The mice were treated with lipopolysaccharide (LPS)- administered intraperitonially at a concentration of 2mg/kg dissolved in 100 µl saline. Saline treated mice served as controls. Ct-values of GAPDH, CXCL1 and CXCL2, CXCL5, CXCL8 and CXCL10 are presented. Both treated and control animals were sacrificed at 2, 4, 6, 12 and 24 h after injections. Results represent mean ± SEM values of three experiments (in duplicate).

	**WT**	**IL6-KO**	**WT**	**IL6-KO**	**WT**	**IL6-KO**
Time (h)	GAPDH		CXCL1		CXCL2	
Control	15.0±0.2	15.6±0.1	27.6±0.1	28.8±0.3	34.9±0.2	36.0±0.5
2	15.7±0.3	15.6±0.2	20.7±0.2	21.6±0.2	23.7±0.2	24.2±0.3
4	16.1±0.5	15.9±0.2	21. ±0.2	24.3±0.5	26.7±0.3	28.5 ±0.2
6	16.1±0.1	15.4±0.1	21.7±0.2	23.8±0.6	26.8±0.2	28.3±0.1
12	15.3±0.2	14.8±0.1	22.1±0.3	24.7±0.2	27.4±0.3	29.1±0.1
24	15.3±0.1	15. ±0.2	27.2±0.3	28.6±0.1	33.2±0.4	34.1±0.5

	WT	IL6-KO	WT	IL6-KO	WT	IL6-KO
Time (h)	CXCL5		CXCL8		CXCL10	
Control	34.1±2.4	36.9±1	33.4±0.2	35.3±0.6	28.1±0.1	28.1±0.3
2	25.9±0.7	26.9±0.1	27.2±0.2	27.9±0.2	17.2±0.3	17.1±0.4
4	28.3±0.1	28.1±1	27.7±0.4	30.6±0.7	18.9±0.2	19.2±0.5
6	28.3±1	27.3±0.1	27.6±0.4	29.1±0.8	23.1±0.4	22.2±0.2
12	29.2±0.5	30.6±0.4	27.9±0.2	30.9±0.1	23.7±0.3	25.6±0.7
24	32.1±0.4	33.1±0.7	33.6±0.3	34.0±0.1	26.8±0.4	26.1±0.2

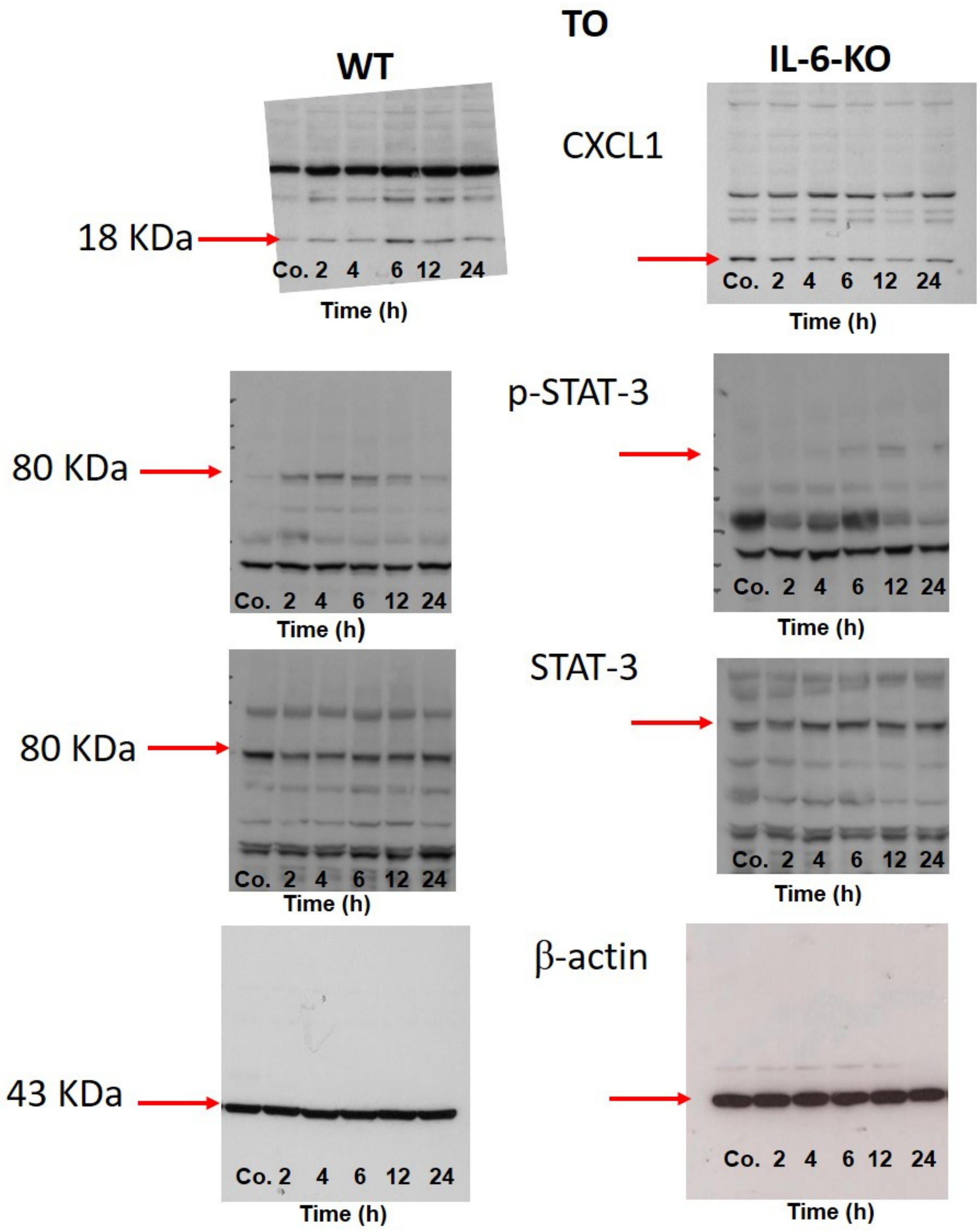

Figure S1: Western blot analyses of protein from mouse liver with antibodies against CXCL1, pSTAT-3 and STAT-3 in wild type and IL6-KO mice. β-actin served as a loading control. The mice were treated with turpentine oil (TO)-administered intramuscularly into the right and left hindlimb of mice at a concentration of 5 ml/kg/each hindlimb. Saline treated mice served as controls. Both treated and control animals were sacrificed at 2, 4, 6, 12 and 24 hours after injections (*n*=3).

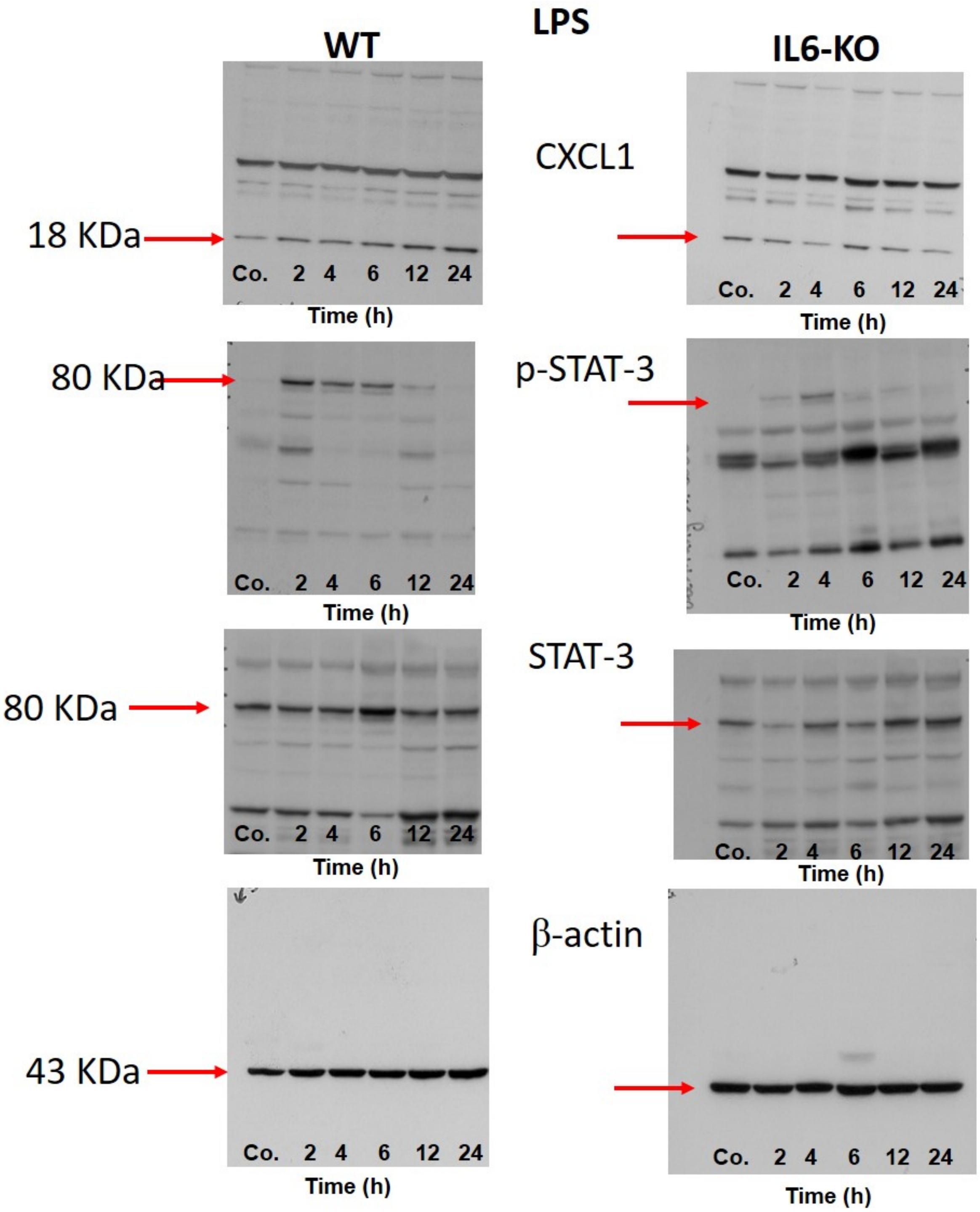

Figure S2: Western blot analyses of protein from mouse liver with antibodies against CXCL1, pSTAT-3 and STAT-3 in wild type and IL6-KO mice. β-actin served as a loading control. The mice were treated with lipopolysaccharide (LPS)- administered intraperitonially at a concentration of 2mg/kg dissolved in 100 µl saline. Saline treated mice served as controls. Both treated and control animals were sacrificed at 2, 4, 6, 12 and 24 hours after injections
